# Severe Atopic Dermatitis: Clinical Confusion With Hyper IgE Syndrome

**DOI:** 10.7759/cureus.79976

**Published:** 2025-03-03

**Authors:** Reyhan S Cansunar, Güzin Özden, Leyla Cevirme, Susamber Dik, Merve Erkoc, Hakan Basir

**Affiliations:** 1 Allergy and Immunology, Adana City Training and Research Hospital, Adana, TUR; 2 Allergy and Immunology, Adana City Training and research Hospital, Adana, TUR

**Keywords:** abrocitinib, atopic dermatitis, hyperimmunoglobuline e syndrome, immunedeficiency, jak inhibitor

## Abstract

Hyper IgE syndrome (HIES) is a rare primary immunodeficiency characterized by chronic eczema, recurrent staphylococcal infections on the skin and pulmonary system, and high serum IgE concentrations. The first clinical sign of HIES is eczema, usually in early infancy. Severe atopic dermatitis (AD) may also mimic HIES with findings of eczema, high serum immunoglobulin E levels, and eosinophilia. Therefore, differential diagnosis may be difficult.

Here, we present a case with eczematoid skin rashes, asthma, elevated serum IgE levels, and skin infections that started in infancy and were followed for nine years by the pediatric allergy and immunology clinic with the diagnosis of HIES, but in fact, had severe AD. Because the patient had no recurrent infections that would suggest immune deficiency during his clinical follow-up, other than the skin infections at the time of diagnosis. In addition, the patient had no non-immunological symptoms of hyper IgE syndrome.

## Introduction

Hyper-IgE syndrome (HIES) is a rare primary immunodeficiency disorder (PID) characterized by chronic eczema, recurrent staphylococcal infections affecting the skin and lungs, and markedly high serum IgE concentrations. This disease was first described in 1966 by Davis and Wedgwood in two girls suffering from recurrent 'cold' staphylococcal abscesses, pneumonia, and neonatal-onset eczematoid rashes [1]. Although most cases are sporadic, autosomal dominant and autosomal recessive cases have also been reported. The first clinical symptom of HIES is eczema, which usually occurs in early infancy. In addition, staphylococcal skin abscesses are called 'cold abscesses' because they do not show signs of inflammation, such as redness or increased temperature [2]. In addition to the classical triad of skeletal, vascular, and connective tissue and dental abnormalities, neurocognitive disorders and viral skin infections can also be seen in some types of HIES [3]. 

Atopic dermatitis (AD) is considered a cardinal feature of immunodeficiency disorders and occasionally the presenting manifestation in various PIDs, including HIES; thus, recognition of distinct features ascribed to HIES can facilitate early diagnosis. Severe AD may also mimic HIES with eczema, high serum IgE levels, and eosinophilia findings, and therefore, differential diagnosis may be difficult [4]. In comprehensive cohort studies, clinical and immunological findings that distinguish HIES from severe AD are being investigated.

## Case presentation

An 18-year-old male patient has been followed up in the Department of Pediatric Allergy and Immunology since he was nine years old. He was referred to our Adult Clinical Allergy and Immunology Department for continued adult follow-up and treatment. Our patient was born at term, with low birth weight via normal vaginal delivery, and was taken into neonatal intensive care for an unspecified duration.

His skin has had widespread redness and eczematous rash since birth, and his diet has been adjusted. The patient was weaned from breastfeeding and given an appropriately formulated baby food for two years while also following a nutritional diet. Various treatments such as inhaled and intranasal steroids, montelukast, antihistamines, and tacrolimus monohydrate were applied until the age of nine with the diagnosis of allergic asthma, rhinoconjunctivitis, and eczema. However, the patient's clinical complaints never fully regressed. At the age of nine, he was hospitalized in the Pediatric Allergy and Immunology Clinic with complaints of increased widespread skin lesions and a fever reaching 38.3°C, and intravenous antibiotic therapy was started. No systemic infectious focus was identified apart from the infectious findings in the skin lesions, and *Acinetobacter baumannii* and *Staphylococcus haemolyticus* growth was detected in the wound cultures. The skin biopsy result was evaluated as spongiotic dermatitis, and lymphocytic infiltration, macrophages, and collagen coarsening were observed in the perivascular area. Laboratory tests performed on the patient at that time revealed IgA: 73 mg/dl (45-250 mg/dl), IgG: 1198 mg/dl (700-1600 mg/dl), IgM: 47 mg/dl (50-250 mg/dl), eosinophil count 2250/μL (0 - 500/μL), C-reactive protein (CRP) 0.38 mg/dl (0 - 0.8 mg/dl), and total IgE level was 29300 IU/ml (10-180 IU/ml) (Table 1). Atopy tests were also performed, and specific IgE levels were found to be positive for cow's milk (high positive), wheat flour (strong positive), nut mix (strong positive), and egg white (positive). From this period onwards, intravenous immunoglobulin (IVIG) treatment at a dose of 0.5 g/kg was started to be given IV monthly with the diagnosis of HIES. There was no consanguinity between the parents in the family history; his mother had similar disease characteristics. Genetic tests were done. The dedicator of cytokinesis 8 (DOCK8) and whole exome sequencing (WES) analysis were reported as normal. Differential diagnosis is quite difficult because of the significant mimicry between HIES and AD. During long-term follow-up, diagnoses can be made as clinical and laboratory findings of diseases occur.

**Table 1:** Blood test results WBC count: White blood cell count; %: Percentage; ESR: Erythrocyte sedimentation rate; CRP: C reactive protein; IgE: Immunoglobulin; IgA: Immunoglobulin A; IgM: Immunoglobulin M; IgG: Immunoglobulin G; g/dL: Gram per deciliter; mg/dL: Milligrams per deciliter; g/L: Grams per liter; mm/h: Millimeters per hour; IU/mL: International unit per milliliter

Test	Result (in 2024)	Previous Result (in 2015)	Reference range
WBC count	7400/μL	9200/μL	4500-10300/μL
Neutrophils	2760/μL	3630 /μL	1700 – 7600/μL
Lymphocytes	3460/μL	2830/μL	1000 – 3200/μL
Eosinophils	650/μL	2250/μL	0 - 500/μL
Haemoglobin	16 g/dL	14.5 g/dL	12.5-16.3 g/dL
Haematocrit	48.6%	42.3%	36.2 – 46.3%
ESR	4 mm/h	7 mm/h	0 – 15 mm/h
CRP	1.9 mg/dL	0.38 mg/dL	0 – 5 mg/dL
IgE	271 IU/mL	29300 IU/mL	5 – 160 IU/mL
IgG	15.3 g/L	1198 mg/dL	7 – 16 g/L (2024), 700 – 1600 mg/dL (2015)
IgA	1.56 g/L	73 mg/dL	0.7 – 4 g/L (2024), 45 – 250 mg/dL (2015)
IgM	0.37 g/L	47 mg/dL	0.4 – 2.3 g/L (2024), 50-250 mg/dL (2015)

The patient had a history of hospitalizations for fever, cellulitis findings, and neck abscesses three times until the age of 14, with the longest hospitalization time being three weeks. The last hospitalization was at the age of 14, and imaging performed at that time revealed cellulitis at the left occipital and suboccipital levels, inflamed lymphadenopathies, and bilateral cervical lymphadenomegaly. There was no history of hospitalization afterward.

There were no infectious findings when the patient presented to us, but skin redness, itching, and eczematous rashes persisted. The patient had been using weekly subcutaneous immunoglobulin for four years since the date of his initial HIES diagnosis. Additionally, cyclosporine 100 mg/day had been initiated five months prior to the patient's presentation to our clinic. When he visited our clinic, a detailed physical examination and laboratory tests were performed. There were no physical examination findings other than widespread erythematous, scaly, and pruritic eczematous skin findings. Laboratory tests revealed IgA: 1.56 g/L (0.7-4 g/L), IgG: 15.3 g/L (7-16 g/L), IgM: 0.37 g/L (0.4-2.3 g/L), total IgE level: 271 IU/ml (5-160 IU/ml), eosinophil count 650/μL (0-500/μL), erythrocyte sedimentation rate 4 mm/h (0-15 mm/h), CRP 1.9 mg/L (0-5 mg/dl) (Table 1). The current results indicated a decrease in serum IgE levels. We evaluated that the change in the patient's IgE levels could be attributed to cyclosporine treatment and variations between laboratories.

IVIG treatment was terminated because the patient had no history or clinical findings supporting the diagnosis of immunodeficiency. In addition, cyclosporine treatment was discontinued because the symptoms of AD continued to be severe despite using it for five months. A dermatology consultation was requested to rearrange the treatment of AD, and Abrocitinib treatment was started. The patient has been receiving Abrocitinib 200 mg/day treatment for three months, and his skin lesions have regressed, and he has no active complaints.

## Discussion

HIES is a rare primary immunodeficiency disease with multisystem involvement. There are immunological and non-immunological symptoms. The most common immunological abnormalities are eczematoid skin rashes, skin abscesses, recurrent respiratory tract infections, markedly elevated serum IgE levels, mucocutaneous candidiasis, and eosinophilia. Non-immunological manifestations include craniofacial, musculoskeletal, dental, and vascular abnormalities. In genetic studies, signal transducer and activator of transcription 3 (STAT3), dedicator of cytokinesis 8 (DOCK8), human epidermal growth factor receptor 2 gene-erbb2-interacting protein (ERBIN), caspase recruitment domain family member 11 (CARD11), phosphoglucomutase-3 (PGM3), interleukin 6 (IL6), serine protease inhibitor Kazal-type 5 (SPINK5), transforming growth factor beta receptor 1-2 (TGFBR1-2) mutations have been identified [2,5].

AD, also known as atopic eczema and included in the HIES triad, is a chronic recurrent inflammatory dermatosis closely associated with symptoms of itching and dryness and IgE-mediated sensitivity to aeroallergens and foods. More than 60% of children diagnosed with AD are at risk of developing allergic rhinitis or asthma; this condition is called the 'atopic march' [6].

AD affects 13% of children and approximately 7% of adults in the United States. Childhood-onset AD begins early in life, with 50% diagnosed in the first year of life and 85% by 5 years of age. Although AD often resolves during childhood, it persists through adulthood in 20% to 50% of patients [7]. In infants, lesions tend to be more common on the cheeks and scalp, while in children, the extremities, cheeks, forehead, and neck areas are usually affected. In adolescents and adults, lesions are located in the head and neck region as well as in flexural regions such as the antecubital and popliteal fossa. Diagnosis is based on history and physical examination. The biopsy will show a spongiosa appearance (intracellular edema in the epidermal layer) and a perivascular infiltrate composed of monocytes, lymphocytes, and occasionally eosinophils [6]. The skin biopsy result taken from our patient was reported as spongiotic dermatitis.

Laboratory examinations reveal that serum IgE concentrations and eosinophil levels are increased, especially in asthmatic children. Skin prick and specific IgE tests for suspected or common allergenic foods can yield a positivity rate of 50% [8,9]. Our patient's blood tests at the time of his first diagnosis showed a significant increase in serum IgE levels. We thought that these results would guide the diagnosis of HIES. In our patient specifically, IgE testing was positive for milk, flour, nuts, and eggs.

The differential diagnosis includes seborrheic dermatitis, contact dermatitis, psoriasis, pityriasis rosea, and various infective and neoplastic conditions. AD may also be a component of various immunodeficiencies, such as HIES, Wiscott-Aldrich syndrome, and severe combined immunodeficiency. Skin superinfection, especially with Staphylococcus Aureus, is also quite common in patients with AD. Our patient's infective findings during childhood were also in the form of skin infection, and there was no history of recurrent pulmonary infection. This situation led us away from the diagnosis of HIES.

Scoring systems have been developed to assess the severity of skin lesions in AD objectively. SCORing Atopic Dermatitis (SCORAD) was created for this purpose; it includes subjective and objective parameters for evaluation: subjective criteria are itching and sleep disturbance, and objective ones evaluate the extent and intensity of injuries. Dermatitis is considered severe when the SCORAD score is > 50 [10]. Our patient had severe AD, according to the SCORAD index. The National Health Institute (NIH) HIES score was also calculated to assist in differential diagnosis; the result was found to be 33. (< 20 points = HIES is unlikely, 20 - 40 points = questionable score, > 40 points = in favor of HIES). In a patient with high IgE levels, the main clues that will lead to a diagnosis of primary immune deficiency include severe eczema, a history of frequent infections, connective tissue and skeletal system anomalies or syndromic appearance, and the presence of autoimmunity. There were no clinical findings other than severe eczema in our patient. In such a case, clinicians should carefully evaluate whether there is any evidence of infection other than skin infections complicating AD.

In patients with severe AD who do not respond to effective treatments and have additional clinical symptoms suggestive of immune deficiency, a detailed evaluation should be performed for the diagnosis of HIES. However, it should be kept in mind that when AD is severe, as in this case report, these two diseases can be confused with each other with clinical and laboratory findings. Patients should be evaluated for symptoms and signs other than AD during their clinical follow-up.

The first step in AD treatment involves avoiding triggers and restoring the epidermal barrier with moisturizers, pruritus control, and topical steroids. Immunosuppressive drugs such as oral corticosteroids, cyclosporine A, mycophenolate mofetil, and methotrexate may also be helpful, but their use is limited due to their side effects [11,12]. Dupilumab is a biologic agent used to treat adults with moderate to severe AD [13]. Janus kinase (JAK) inhibitors abrocitinib and upadacitinib also act by reducing proinflammatory cytokines through inhibition of the Janus kinase/signal transducer and activator of transcription (JAK-STAT) pathway and are approved for use in moderate to severe AD [14].

## Conclusions

As a result, AD in HIES is a serious and challenging condition. HIES can sometimes be clinically indistinguishable from severe AD. Our patient had features of severe AD and high serum IgE levels but no dysmorphic features or recurrent severe infections, which complicated the differential diagnosis of HIES. Moreover, genetic tests for HIES were negative.

Moderate to severe AD should be treated regardless of the presence of immunodeficiency. In recent years, JAK inhibitors, which have been utilized in treating AD, have shown promising results. In our patient, the symptoms of AD have regressed following three months of Abrocitinib treatment. However, our clinic will closely monitor the patient for potential side effects of the treatment and any new clinical findings that may arise during follow-up.
